# Measuring Spinal Cord Potentials and Cortico-Spinal Interactions After Wrist Movements Induced by Neuromuscular Electrical Stimulation

**DOI:** 10.3389/fnhum.2022.858873

**Published:** 2022-03-11

**Authors:** Michael Wimmer, Kyriaki Kostoglou, Gernot R. Müller-Putz

**Affiliations:** ^1^Institute of Neural Engineering, Graz University of Technology, Graz, Austria; ^2^BioTechMed-Graz, Graz, Austria

**Keywords:** electroencephalogram, electrical stimulation, movement, sensorimotor areas, somatosensory evoked potentials, spinal cord potentials, directed coherence, generalized partial directed coherence

## Abstract

Electroencephalographic (EEG) correlates of movement have been studied extensively over many years. In the present work, we focus on investigating neural correlates that originate from the spine and study their connectivity to corresponding signals from the sensorimotor cortex using multivariate autoregressive (MVAR) models. To study cortico-spinal interactions, we simultaneously measured spinal cord potentials (SCPs) and somatosensory evoked potentials (SEPs) of wrist movements elicited by neuromuscular electrical stimulation. We identified directional connections between spine and cortex during both the extension and flexion of the wrist using only non-invasive recording techniques. Our connectivity estimation results are in alignment with various studies investigating correlates of movement, i.e., we found the contralateral side of the sensorimotor cortex to be the main sink of information as well as the spine to be the main source of it. Both types of movement could also be clearly identified in the time-domain signals.

## Introduction

Recording and investigating neural correlates of movement or sensation with non-invasive electroencephalography (EEG) has become standard practice over the past years. Since the introduction of modern artifact reduction or detection algorithms in online settings, clean EEG can be derived for a great variety of applications. However, not only potentials and patterns recorded from the scalp are of interest, studying the development and propagation of potentials in the spinal cord could be interesting as well and potentially lead to new insights.

Numerous studies have established the usefulness of recording spinal cord potentials (SCPs) for clinical applications, namely as a monitoring tool during surgical operations as well as an indicator of spinal diseases.

Multiple attempts to monitor the spinal cord intraoperatively with somatosensory evoked potentials (SEPs) recorded from the scalp have shown to be strongly influenced by the preceding anesthesia and are therefore insufficient for that purpose (Levy et al., 1984; Grundy and Villani, 1988). However, Macon et al. (1982) and Macon and Poletti (1982) showed that measuring the spinal cord conduction velocity *via* epidural electrodes is a reliable and safe technique to provide physiological means of monitoring spinal cord functions during spinal operations and is practically unaffected by anesthetic agents. Tani et al. (1995) showed that patients suffering from spinal conduction block, resulting from a cervical spondylotic myelopathy (compression of the spinal cord), have SCPs with a distinctly different waveform, i.e., a reduction in amplitude. With the guidance of evoked SCPs, the level of the conduction block in the spinal cord could be revealed intraoperatively and was closely related to the corresponding compression level from magnetic resonance imaging (MRI) recordings. Among further applications, SCPs were also successfully used during aortic surgery to prevent postoperative paraplegia (Kano et al., 1995).

Measuring SCPs is a promising aid in the diagnosis of spinal cord diseases (Yamada et al., 2004). In a study with six patients with typical amyotrophic lateral sclerosis (ALS) syndrome symptoms (e.g., muscle atrophy, muscle weakness or muscle twitch, among others), no slow, neither positive nor negative potentials could be detected (Shimizu et al., 1979a,b). In patients with spinal tumors a complete block of nerve conduction along the spinal cord after electrical stimulation of the cauda equina cord was found (Shimizu and Shimoji, 2006).

In response to the stimulation of a peripheral nerve or root, segmentally evoked SCPs can be measured from the same or nearby segment. For example, Shimoji et al. (1972) recorded SCPs on the cervical spinal cord after stimulation of the ulnar nerve. The segmentally evoked SCP waveform typically consists of an initial positive spike (P1), followed by a sharp negative (N1) and a second positive wave (P2). Although most of the studies recorded SCPs from the epidural space with needle electrodes, very similar patterns were also found using skin surface electrodes (Shimoji et al., 1978). Since SCPs have been extensively studied, there is a general agreement on the biological origins of the fundamental pattern; several experiments (Bernhard, 1953; Fernandez de Molina and Gray, 1957) substantiated that the first positivity (P1) reflects the action potential propagating along primary afferent neurons. The following sharp negativity (N1) is agreed to be produced by neurons of at least second-order. Primarily the activity of interneurons is described to cause the N1 wave (Eccles and Sherrington, 1930; Coombs et al., 1956). The second positivity (P2) is thought to be closely related to the process of primary afferent depolarization (PAD), a mechanism causing presynaptic inhibition alongside primary sensory afferents (Koketsu, 1956; Eccles et al., 1963). *Via* intracellular recordings in primary afferent fibers it was shown that the time course of PAD closely correlates to the course of the positive wave P2.

Sensory information related to proprioception or tactile sensations is transmitted by the dorsal column-medial lemniscal system (Kandel et al., 2000). This information from the upper limbs ascends ipsilaterally in the spine by first-order neurons that form the cuneate fasciculus and terminate in the cuneate nucleus in the medulla. After the sensory decussation, the information ascends through second-order neurons that form the medial lemniscus and terminate at the ventral posterolateral nucleus of the thalamus. Finally, thalamic neurons relay the information to the somatosensory cortex in the postcentral gyrus, where the related electrical activity can be measured, e.g., using non-invasive recording techniques.

Herein, our main goal was to study the generation and the interaction of SCPs and EEG signals during wrist movements, namely a wrist extension induced by functional electrical stimulation (FES) and a subsequent flexion caused by the termination of the stimulation. FES was preferred over an active movement in order to assess the response of only afferent signals during the movement execution period. Hence, possible inferences of afferent and efferent signals were prevented.

We hypothesized that using only EEG and SCPs we can non-invasively identify a directional connection from the spinal cord to the cortex. The wrist movement is expected to cause the information to flow to the contralateral side of the primary motor cortex. Moreover, sensory input in the hand and fingers after the termination of the induced movement are expected to cause somatosensory areas to be additional sinks of information. To study these connections we used directed coherence (DC) (Baccalá et al., 1998) and generalized partial directed coherence (GPDC) (Baccala et al., 2007) as multivariate estimators of directedness in the frequency domain. DC and GPDC are based on the concept of Granger causality (Granger, 1969) and are computed using the multivariate autoregressive (MVAR) model framework (Blinowska et al., 2004). MVAR models have been previously applied to capture causality in various physiological systems and especially in brain dynamics (both physiological and pathological). Cortico-cortical couplings (based on EEG signals) are usually the main topic of interest (Anderson et al., 1998; Möller et al., 2001; Kus et al., 2004; Schlögl and Supp, 2006; Astolfi et al., 2007, 2009a; Babiloni et al., 2009; Hu et al., 2012; Siggiridou and Kugiumtzis, 2016). However, there is a large body of literature that focuses on brain cross-talks (e.g., cortico-muscular interactions based on EEG and magnetoencephalography signals) (Fang et al., 2009; Xifra-Porxas et al., 2018). To the best of our knowledge, this paper is the first to study cortico-spinal interactions using MVAR models. For the first time, we were able to simultaneously measure FES-induced SCPs along with EEG signals over sensorimotor areas. The recorded SCPs and EEG signals were then used to estimate MVAR models and extract DC and GPDC values in order to quantify the strength and the directionality of the information flow from spine to brain and vice versa.

## Materials and Methods

### Participants and Experimental Procedure

Ten healthy right-handed participants (six males, four females; age 25.4 ± 3.7 years, mean ± *SD*) took part in the present study. After a detailed oral and written instruction, all participants gave written informed consent to participate in the study.

**FIGURE 1:**
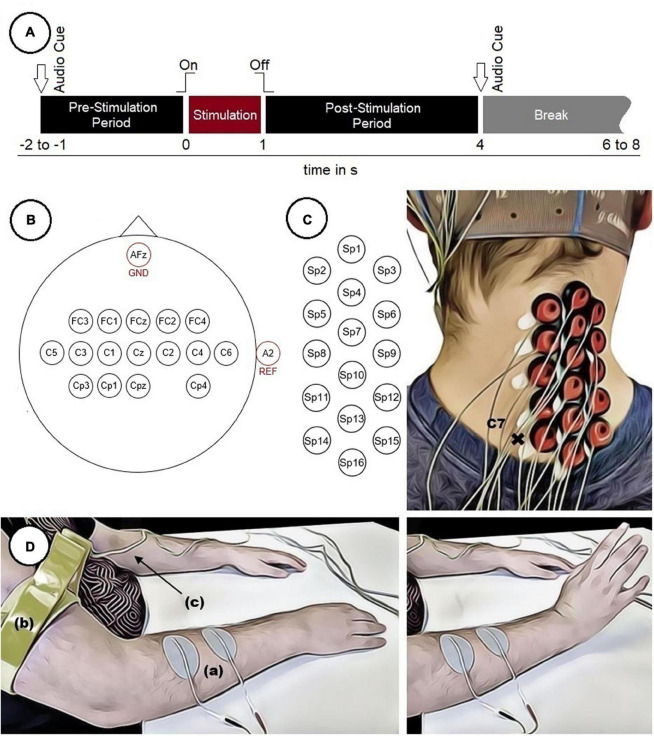
Experimental setup. **(A)** Timing of a single trial. **(B)** Electrode positions on the scalp. **(C)** Electrode positions on the neck used to measure SCPs with marked vertebra C7. **(D)** Participant with two surface electrodes **(a)** for electrical stimulation and a limb clamp **(b)** with potential equalization **(c)** performing a FES-induced wrist movement.

In FES, electrical stimulation is applied to peripheral motor neurons to cause muscles to contract. The strength of the muscle contraction can be regulated by varying the stimulation current and stimulation frequency (Peckham and Knutson, 2005). In the present work, FES was used to cause a wrist movement as depicted in Figure 1D.

Before the start of the experiment, two surface electrodes (oval, 4 cm × 6.4 cm) were positioned on the right forearm above the wrist extensor muscles (in particular the extensor carpi radialis longus and brevis muscles) to apply the stimulation current. For each participant an individual stimulation current (16.0 ± 4.1 mA, mean ± *SD*) was used. An appropriate stimulation current was found by gradually increasing it until the maximum wrist extension possible was reached without causing any pain to the participants. Additionally, the stimulation electrodes were repositioned until the movement was executed in a comparable manner in all participants. The FES was executed with a Microstim 8 stimulator (Krauth+Timmermann, Hamburg, Germany). The stimulation frequency was set at 35 Hz for all participants, using a biphasic constant current pulse with a pulse width of 300 μs. To reduce the influence of artifacts related to the stimulation, a limb clamp electrode was attached to the right upper arm of each participant and connected to the potential equalization of the biosignal amplifier.

Each participant performed 240 movements (8 runs × 30 trials). Each trial started with a pre-stimulation period (1–2 s, randomized) and ended with a post-stimulation period (3 s) in which the participants were not asked to perform any specific task. The duration of the stimulation was 1 s. Audio cues indicated the beginning (high beep tone) and the end (low beep tone) of the trials. The intertrial interval (break) was also randomized (2–4 s) to reduce both the possibility to predict the stimulation onset and the participant’s temporal adjustment to the paradigm. This resulted in a total length of about 9 s per trial plus intertrial interval and an overall duration of about 35 min for the whole experiment. This excludes breaks of about 2–5 min between runs to prevent muscle fatigue in the forearm and to relax the muscles in the neck. The timing of a single trial is depicted in Figure 1A. During the experiment the participants were seated comfortably in an armchair and were instructed to fixate their eyes on a cross (approximately 1.5 m in front of them as well as to restrict movements like swallowing to the intertrial interval.

### Signal Recordings

EEG was recorded both over cortical areas of the brain and the right side of the cervical spinal cord (ipsilateral to the stimulation site). Sixteen electrodes placed over cortical areas were positioned according to the 10–10 system (Nuwer et al., 1998), covering mainly sensorimotor areas (Figure 1B). To measure SCPs, 16 electrodes were positioned from the vertebrae C7/Th1 upwards with a distance of approximately 18 mm between two neighboring electrodes (center to center) (Figure 1C). Therefore, the vertebra prominens that has a distinctive spinous process usually palpable from the skin surface, was marked as an indicator to position the electrodes in a consistent manner throughout all participants. The ground electrode was placed at position AFz and the reference electrode at the right ear lobe.

The recording of all signals was performed with the g.GAMMAsys system with g.LADYbird active electrodes and two g.USBamp biosignal amplifiers (Guger Technologies, Graz, Austria). The signals were filtered from 0.1 to 100 Hz using an 8th order Chebyshev filter and a notch filter was applied at a center frequency of 50 Hz to suppress power line interference. The sampling rate was 512 Hz. MATLAB R2015a and Simulink 8.0 (The MathWorks, Massachusetts, United States) were used to record the data and TOBI SignalServer (Breitwieser et al., 2010) for data acquisition.

### Signal Processing

The recorded signals were processed offline using MATLAB R2019b (The MathWorks, Massachusetts, United States) with the EEGLAB toolbox (Delorme and Makeig, 2004), the BioSig toolbox (Schlögl and Brunner, 2008) and the eMVAR toolbox (Faes et al., 2013).

We filtered the raw data between 0.5 and 60 Hz (zero-phase 5th order high-pass and zero-phase 3rd order low-pass Butterworth filter) and applied a notch filter at the stimulation frequency of 35 Hz. The filtered data was then segmented into epochs of 6.5 s (from 2.5 s prior to stimulation onset until 4 s post stimulation). Corrupted epochs were automatically rejected based on amplitude thresholding, as well as abnormal joint probabilities and kurtosis, similar to Ofner et al. (2019). For the latter two we set the threshold to five and four times the standard derivation, respectively. Subsequently, we rejected contaminated epochs based on visual inspection. On average we used 85% of the trials per participant for further analysis. This is equivalent to more than 2,000 trials used to calculate grand average results.

Thereafter, we performed independent component analysis (ICA) (Makeig et al., 1996) by applying the extended Infomax algorithm (Lee et al., 1999) implemented in the EEGLAB toolbox. Based on visual inspection, we rejected independent components that corresponded either to biological artifacts (e.g., heartbeat, muscle or eye movements, blinks) or the electrical stimulation itself.

### Measuring Directional Connectivity

To examine the directional connectivity between EEG and SCPs we applied the multivariate autoregressive model (MVAR) methodology on the grand average signals (over all subjects). Suppose ***y***(*n*) is a vector containing the samples of the *M* channels at time point *n* (where *M* = 32, i.e., 16 EEG and 16 SCP channels). The corresponding MVAR model is described as,


(1) $$\text{y}\,\left(n\right)=\sum_{k=1}^{p}{\text{A}}_{k}\,\text{y}\,\left(n-k\right)+\varepsilon \,\left(n\right)$$

where *p* is the model order, **A_k_** ∈ **R**^*M*×*M*^ contains the autoregressive coefficients for each order *k* and ε(*n*) ∈ **R**^*M*×1^ denotes the uncorrelated error following a Gaussian distribution with zero mean. Equation 1 describes the dynamic effect of each time series to itself but also to all other time series. To estimate the MVAR coefficients **A_k_**, an important step is the selection of an appropriate model order (*p*). Herein, *p* was optimized using the Akaike Information Criterion (AIC) (Akaike, 1974), which aims to minimize both the model error and the model order (i.e., model complexity) (Kostoglou et al., 2019),


(2) $$A\,I\,C\,\left(p\right)=N\,l\,o\,g\,\left(\left|\hat{\Sigma }\right|\right)+2\,C$$

where *N* is the total number of samples, $\left|\hat{\Sigma }\right|$ is the determinant of the covariance of the residual errors (i.e., $\hat{\Sigma }=c\,o\,v\,\left(\text{Y}-\hat{\text{Y}}\right)$; whereby **Y** represents the actual multivariate time series and $\hat{\text{Y}}$ the predicted ones based on the MVAR model) and *C* = *M*^2^*p* the total number of MVAR coefficients.

Transforming Eq. 1 to the frequency domain yields **Y**(*f*) = **H**(*f*) **E**(*f*), where the transfer matrix **H**(*f*) is defined as $\text{H}\,\left(f\right)={\left[\text{I}-\text{A}\,\left(f\right)\right]}^{-1}=\underline{\text{A}}\,{\left(f\right)}^{-1}$ and $\text{A}\,\left(f\right)=\sum_{k=1}^{p}{\text{A}}_{k}\,e^{-i\,2\,\pi \,f\,k\,T}$is the coefficient matrix (**I** being the identity matrix). The elements of the transfer function and coefficient matrices can be used to assess directional couplings between the different time series under consideration. Baccalá et al. (1998) and Baccala et al. (2007) proposed the concepts of directed coherence (DC) and GPDC in order to study, in the frequency domain, the directional influences of any given pair of channels in a multivariate data set. DC quantifies both direct and indirect causal links between two time series, while GPDC considers only the direct paths of information flow. The DC and GPDC from time series *d* (driver) to time series *t* (target) at frequency *f* is defined as,


(3) $$D\,C_{t\,d}\,\left(f\right)=\frac{\sigma_{d}\,H_{t\,d}\,\left(f\right)}{\sqrt{\sum_{m=1}^{M}\sigma_{m}^{2}\,{\left|H_{t\,m}\,\left(f\right)\right|}^{2}}}$$

(4) $$G\,P\,D\,C_{t\,d}\,\left(f\right)=\frac{\frac{1}{\sigma_{T}}\,{\underline{A}}_{t\,d}\,\left(f\right)}{\sqrt{\sum_{m=1}^{M}\frac{1}{\sigma_{m}^{2}}\,{\left|{\underline{A}}_{m\,d}\,\left(f\right)\right|}^{2}}}$$

with $\Sigma =d\,i\,a\,g\,\left(\sigma_{1}^{2}\,\ldots \,\sigma_{M}^{2}\right)$being the diagonal covariance matrix of ε. Since **Σ** is not known in practice, an estimate $\hat{\Sigma }$ can be obtained from the model residuals. Note that DC is normalized to show the ratio between the flow of information from channel *d* to channel *t* to all the flows toward target channel *t* (at frequency *f*), whereas GPDC is normalized to show the ratio between the flow from channel *d* to channel *t* to all the flows originating from driver channel *d.* Therefore, DC emphasizes mainly the sources and GPDC the sinks of information (Blinowska, 2010).

To evaluate channel importance, the total information outflow from a particular channel was defined as the sum of statistically significant DC values toward all other channels (Astolfi et al., 2007). On the other hand, the total information inflow to a particular channel was defined as the sum of statistically significant GPDC values from all other channels,


(5) $$O\,u\,t\,f\,l\,o\,w_{d}\,\left(f\right)=\sum_{t=1,t\neq d}^{M}D\,C_{t\,d}^{*}\,\left(f\right)$$

(6) $$I\,n\,f\,l\,o\,w_{t}\,\left(f\right)=\sum_{d=1,d\neq t}^{M}G\,P\,D\,C_{t\,d}^{*}\,\left(f\right)$$

where $D\,C_{t\,d}^{*}\,\left(f\right)$ and $G\,P\,D\,C_{t\,d}^{*}\,\left(f\right)$ refer to statistically significant DC and GPDC values (from time series *d* to time series *t*), respectively.

To evaluate statistical significance, we generated surrogate multivariate signals (Faes et al., 2010) and estimated MVAR models. With the surrogate data we eliminated possible causal interactions between different channels and generated a reference DC/GPDC distribution under the null hypothesis of no causality from time series *d* to *t*. The significance of the DC/GPDC values evaluated from the actual data was then assessed using the reference DC/GPDC distribution. We considered DC/GPDC values below the 95th percentile from the reference distribution as non-significant and consequently set them to zero. Hence, the corresponding pairs were regarded to have no directional influence on each other.

To study the directional relationship between signals recorded from the spinal cord and cortical areas, we divided the resulting evoked potentials (2 × 16 time courses after averaging all participants) into two parts. The first part concentrates on the wrist extension induced by the FES and contains all samples from the stimulation period (1 s). The second part investigates the information flow during the wrist flexion, which was performed in the absence of any electrical stimulation, and contains the samples from the post-stimulation period only (from the first second after the stimulation offset). We calculated the DC/GPDC for each pair of the 32 signals and for both parts separately in the frequency range of [0.5, 60] Hz.

We further defined the following regions of interest for the head: Fl (frontal left, FC1, FC3), Fz (frontal central, Fz), Fr (frontal right, FC2, FC4), Cl (central left, C1, C3, C5), Cz (central, Cz), Cr (central right, C2, C4, C6), CPl (central parietal left, Cp1, Cp3), CPz (central parietal, Cpz) and CPr (central parietal right, Cp4), and for the neck: ROl (rostral left, Sp2, Sp5), ROz (rostral central, Sp1, Sp4, Sp7), ROr (rostral right, Sp3, Sp6), CAl (caudal left, Sp8, Sp11, Sp14), CAz (caudal central, Sp10, Sp13, Sp16) and CAr (caudal right, Sp9, Sp12, Sp15). Inflow and outflow values for each region were the averages of the values of the corresponding electrodes. The inflow and outflow for regions Fz, Cz and CPz were identical to the ones from the eponymous electrodes.

## Results

### Evoked Response

**FIGURE 2:**
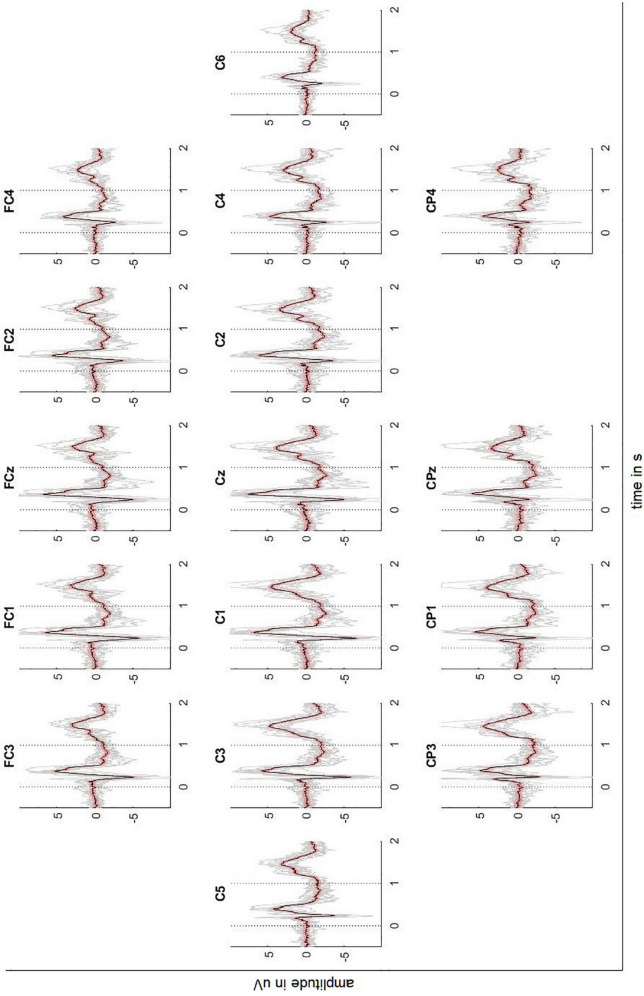
Averaged EEG signals recorded above the sensorimotor cortex with ± 2 standard error (red) and individual results of the ten participants (gray). Dotted lines indicate the stimulus onset and offset.

**FIGURE 3:**
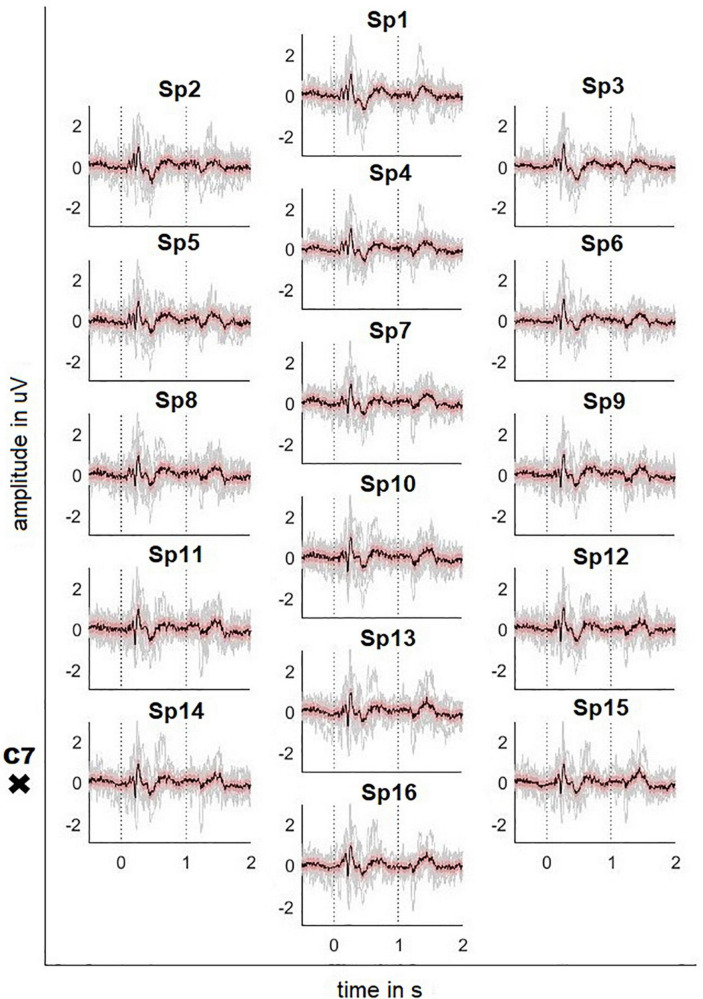
Averaged SCP signals recorded on the neck with ± 2 standard error (red) and individual results of the 10 participants (gray). Dotted lines indicate the stimulus onset and offset. The position of the vertebra prominens is marked with a cross (electrode positions according to Figure 1C).

Figures 2, 3 depict the grand average response (in black) of the signals recorded at both the channels above cortical areas and the spinal cord. The dotted lines indicate the stimulation onset (at *t* = 0 s) and offset (at *t* = 1 s). The variability of the signals is shown in red (±2 standard error) and the individual averages of the participants in gray.

The averaged response of the motor-related brain areas (e.g., electrode position C3) shows a small positivity at *t* = 172 ms after the stimulation onset with a peak amplitude 1.21 μV, followed by a slow negative wave with a peak amplitude -5.99 μV at *t* = 240 ms and by another positivity with a peak amplitude 5.82 μV at *t* = 384 ms. The signal continues to be negative (maximum negativity is -2.42 μV) while the stimulation is on. Thereafter, the signal presents a positivity peaking at 4.81 μV 445 ms after the stimulation offset.

The averaged SCPs show a generally similar pattern, but positive and negative waves appear earlier than in the cortex. For the electrode position Sp6, the first positivity has a peak amplitude 0.32 μV at *t* = 184 ms, followed by a negativity with a peak amplitude -0.48 μV at 218 ms and a second positivity with a peak amplitude 1.04 μV at 269 ms. Finally, the signal shows a broader positivity with a peak amplitude 0.40 μV at *t* = 440 ms after the stimulation offset.

However, the Motionstim 8 delays the electrical stimulation after the stimulation onset by well above 100 ms and therefore, the difference in time from one time point to another is more important than the exact time points of the characteristic waves of the evoked potentials. Table 1 contains the exact timings for the N1 wave and P2 wave as well as their difference in all channels and all defined regions.

**TABLE 1:** Average time (from *t* = 0 s is the stimulation onset) until the N1 wave and P2 wave peak.

Channel		*t*_*N*1_(*s*)	*t*_*P*2_(*s*)	*t*_*P*2_−*t*_*N*1_(*s*)	Region	*t*_*N*1_(*s*)	*t*_*P*2_(*s*)	*t*_*P*2_−*t*_*N*1_(*s*)
EEG	FC3	0.242	0.373	0.130	Fl	0.243	0.370	0.126
	FC1	0.244	0.367	0.123	Fz	0.246	0.365	0.119
	FCz	0.246	0.365	0.119	Fr	0.248	0.377	0.128
	FC2	0.248	0.367	0.119	Cl	0.240	0.384	0.144
	FC4	0.248	0.386	0.138	Cz	0.244	0.371	0.126
	C5	0.242	0.398	0.156	Cr	0.247	0.386	0.138
	C3	0.240	0.384	0.144	CPl	0.246	0.387	0.141
	C1	0.238	0.371	0.132	CPz	0.255	0.384	0.128
	Cz	0.244	0.371	0.126	CPr	0.253	0.396	0.142
	C2	0.246	0.373	0.126				
	C4	0.248	0.388	0.140				
	C6	0.248	0.396	0.148				
	Cp3	0.246	0.388	0.142				
	Cp1	0.246	0.386	0.140				
	Cpz	0.255	0.384	0.128				
	Cp4	0.253	0.396	0.142				
SCP	Sp1	0.220	0.267	0.046	ROl	0.218	0.267	0.048
	Sp2	0.220	0.267	0.046	ROz	0.218	0.268	0.049
	Sp3	0.218	0.267	0.048	ROr	0.218	0.268	0.050
	Sp4	0.218	0.267	0.048	CAl	0.217	0.268	0.050
	Sp5	0.218	0.267	0.048	CAz	0.218	0.268	0.050
	Sp6	0.218	0.269	0.050	CAr	0.217	0.268	0.051
	Sp7	0.216	0.269	0.052				
	Sp8	0.216	0.267	0.050				
	Sp9	0.216	0.269	0.052				
	Sp10	0.216	0.267	0.050				
	Sp11	0.216	0.267	0.050				
	Sp12	0.216	0.267	0.050				
	Sp13	0.218	0.267	0.048				
	Sp14	0.218	0.269	0.050				
	Sp15	0.218	0.269	0.050				
	Sp16	0.218	0.269	0.050				

*Times are calculated from grand average results of the 10 participants for all channels and regions.*

### Directional Connectivity Estimation

**FIGURE 4:**
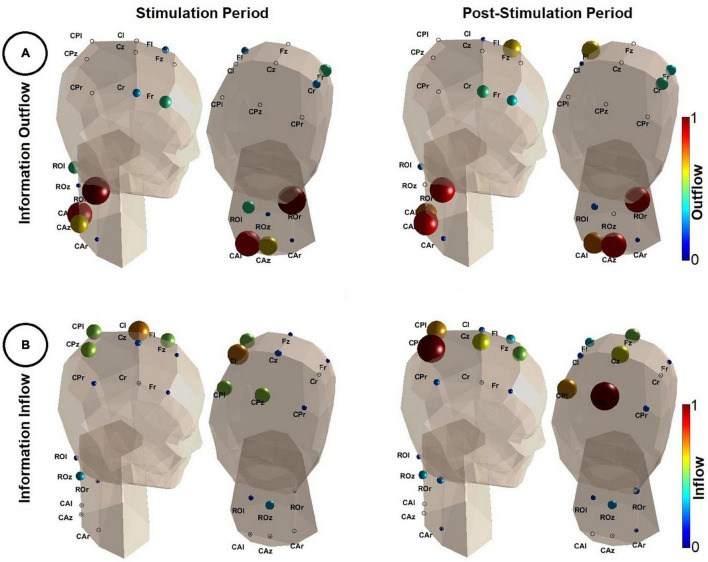
Results of the directional connectivity estimation during the stimulation (*t* = [0, 1] s–left panel) and the post-stimulation period (*t* = [1, 2] s–right panel). **(A)** Depicts the information outflow (Eq. 5) in the frequency range of [0.5, 60] Hz. Note that the color (color coded based on the color bars shown on the right of each image) and the size of each sphere quantifies the amount of information outflow from a specific region. The larger the spheres and the darker their red, the higher the information outflow from the corresponding regions. Outflow values were normalized by the maximum outflow value detected in both analyzed time periods (i.e., stimulation and post-stimulation). The head model is shown from two different angles. **(B)** Depicts the information inflow (Eq. 6). Similarly as in **(A)**, the larger the spheres and the darker their red, the higher the information inflow of the corresponding regions. Inflow values were normalized by the maximum inflow value detected in both analyzed time periods. A 3D animation can be found in Supplementary Material (Video 2.avi for outflow during stimulation, Video 3.avi for inflow during post-stimulation, Video 4.avi for inflow during stimulation and Video 5.avi for outflow during post-stimulation). This figure was inspired by the eConnectome toolbox (He et al., 2011).

The MVAR analysis resulted into MVAR models of order *p* = 8 and *p* = 7 for the stimulation and post-stimulation period, respectively. Figure 4 illustrates a neck and head model (Mayol-Cuevas, 2022) along with the sources and sinks of information (i.e., information outflow and inflow, respectively) in the broad frequency range of [0.5, 60] Hz.

In Figure 4A, during both the stimulation and the post-stimulation period, regions of the spine were the main sources of information. Consistently, ROr was the strongest driver, partially including more caudal parts of the spine (CAl and CAz). Sensorimotor areas had comparably small outflow values and hence appeared to be at best weak sources of information flow.

The results in Figure 4B show a reversed picture. Low inflow values in all regions of the spinal cord indicate little information flow from the brain to the spine. Main sinks of information were the sensorimotor areas, most prominently those residing on the contralateral side. During the stimulation (induced wrist extension), contralateral sensorimotor areas (in particular Cl) were the main target of information flow as well as, to some extent, parietal and frontal regions. After the stimulation offset (wrist flexion, hand falls back on the table) information flow shifted to more parietal regions of the cortex.

## Discussion

In the present study we recorded both EEG signals and non-invasive SCPs as responses to FES-induced wrist movements. Subsequently, we are the first to use non-invasive signals originating from the cortex and the spine to study cortico-spinal interactions with MVAR models. The measured SCPs in the time domain are in alignment with previous studies and reflect their biological origin. Directed connectivity estimators showed an information flow from the spine to the brain, whereby contralateral sensorimotor areas could be identified as main sinks of information.

Subsequently, we could find the spinal response of afferent fibers to a wrist movement in the time domain using surface electrodes and non-invasive recording techniques. The main characteristics of the found time courses of the SCPs (N1 wave, P1 wave and N2 wave) are in alignment with previous studies in which commonly epidural electrodes were used (Shimoji et al., 1972, 1978). Additionally, the response of the wrist flexion (including the hand falling down on a table) could be clearly identified as a positivity in the time domain.

It was possible to show the information flow from the spine to the brain using directed connectivity estimators that are commonly used for studying cortical networks, namely DC in order to find the sources of information and GPDC to find the sinks. MVAR-based measures have been previously used to study the effect of neuromuscular electrical stimulation on EEG connectivity (Astolfi et al., 2009b; Hu et al., 2012; Porcaro et al., 2013; He and Pursiainen, 2021). However, herein we focus on the interactions between and within the brain and the spine. Furthermore, instead of the commonly used Directed Transfer Function (DTF) and Partial Directed Coherence (PDC), we employed DC and GPDC which are generalized definitions of DTF and PDC, respectively. Contrary to DTF and PDC, DC and GPDC share the property of scale invariance (Baccalá et al., 1998; Baccala et al., 2007; see Eqs 3 and 4); the variance of each time series is taken into account, contrary to DTF and PDC where the covariance matrix is set to the identity matrix). Scale invariance is an important attribute when investigating cross-talks and especially in our case where EEG and SCP signals exhibit differences in their amplitude and variance.

Based on the MVAR analysis, the right rostral region of the investigated part of the spine was identified to be the main source of information during both the stimulation (wrist extension) and post-stimulation period (wrist flexion). Peripheral fibers that are responsible for wrist movements enter the spinal cord at this level, in particular the radial nerve as a part of the brachial plexus. Information outflow from more caudal parts of the spinal cord (level C6 to C8, approximately) are also present during both periods. This may reflect the involvement of afferent activity of the hand and fingers, whose associated nerves (in particular the median nerve) enter at this level. To examine in more detail the time evolution of the spinal outflow we applied a time-varying MVAR analysis, whereby (spinal only) outflow changes were tracked using sliding windows (0.2 s length and 0.002 s overlap). We observed that information outflow increased considerably in the rostral right parts of the spine with the onset of the stimulation (see Supplementary Material, Video 1.avi). This implies that the main area of the income of the stimulation was primarily the rostral right spinal region. We therefore conclude that we have evidence that the measurement of non-invasive potentials of the spinal cord in fact shows activity which is in alignment with the anatomical structure below and its neurophysiological function during passive wrist extension.

During the induced wrist movement, the contralateral side of the sensorimotor areas (region Cl in particular) was found to be the main sink of information. This is in alignment with the activity during FES-induced wrist extensions investigated earlier (Müller et al., 2003). Especially central-parietal regions were also found to be additional sinks during the movement execution. After the stimulation offset, information was mainly directed to the central-parietal regions. This is caused by the stronger involvement of the somatosensory cortex due to the sensory input after the termination of the movement, which caused the participants’ hand to fall back on the table.

Due to the proximity of the spine to upper limb muscles, biological signals recorded at the surface of the neck are most prone to artifacts caused by muscle activity or tension. Therefore, great attention must be given to the relaxation of the participant, without which recording quality data is not possible. The stimulation itself might be unfamiliar and uncomfortable for participants as well and hence could cause a loss of relaxation. For this reason, single runs were kept short and accompanied by breaks of up to several minutes between them. Influences of the heart activity are visible in the recorded data, but could be distinctly identified and removed with ICA in all participants.

It is worth mentioning that Brunner et al. (2016) showed that volume conduction influences connectivity measures extracted by EEG sensor space data and therefore suggested the use of the corresponding source signals instead to obtain more accurate results. This is, however, in contrast to the findings of Kaminski and Blinowska (2014), who concluded that a possible effect, if there is one, of volume conduction on estimators such as DC and GPDC is negligible. In an earlier work, Kaminski and Blinowska (2017) considered the influence of volume conduction to some extent, but their fundamental conclusion remained the same, i.e., mixing of cortical sources does not undermine critical connectivity results. Our results corroborate the same since the estimated directional influences are in alignment with known literature. Unfortunately, the main limitation of this study is the low number of EEG channels that render source localization unfeasible. Our future goal is to include more EEG channels and investigate directional interactions in source space. This could provide a clearer picture of the underlying connectivity patterns.

## Conclusion

For the first time, we simultaneously recorded somatosensory evoked potentials and spinal cord potentials as responses to FES-induced wrist movements. The goal of this study was to explore these neural answers in detail and elucidate their underlying couplings. We could show that the SCPs measured from electrodes placed above the cervical spine are in direct relation to SEPs measured from EEG over sensorimotor areas. With this study we show first evidence that SCPs can be studied non-invasively and pave the way for many more investigations that could reveal new insights to the information flow during movements.

## Data Availability Statement

The raw data supporting the conclusions of this article will be made available by the authors, without undue reservation.

## Ethics Statement

The studies involving human participants were reviewed and approved by the Medical University of Graz. The patients/participants provided their written informed consent to participate in this study.

## Author Contributions

MW and GM-P conceived and designed the experiment. GM-P had the idea. MW performed the experiments. MW and KK processed the data and analyzed the data. MW, KK, and GM-P interpreted the data and proofread the manuscript. MW wrote the manuscript. KK and GM-P edited the manuscript. All authors contributed to the article and approved the submitted version.

## Conflict of Interest

The authors declare that the research was conducted in the absence of any commercial or financial relationships that could be construed as a potential conflict of interest.

## Publisher’s Note

All claims expressed in this article are solely those of the authors and do not necessarily represent those of their affiliated organizations, or those of the publisher, the editors and the reviewers. Any product that may be evaluated in this article, or claim that may be made by its manufacturer, is not guaranteed or endorsed by the publisher.
